# Profile of Volatile Compounds of On-Farm Fermented and Dried Cocoa Beans Inoculated with *Saccharomyces cerevisiae* KY794742 and *Pichia kudriavzevii* KY794725

**DOI:** 10.3390/molecules26020344

**Published:** 2021-01-11

**Authors:** Gilson Celso Albuquerque Chagas Junior, Nelson Rosa Ferreira, Eloisa Helena de Aguiar Andrade, Lidiane Diniz do Nascimento, Francilia Campos de Siqueira, Alessandra Santos Lopes

**Affiliations:** 1 Laboratório de Processos Biotecnológicos (LABIOTEC), Programa de Pós-graduação em Ciência e Tecnologia de Alimentos (PPGCTA), Instituto de Tecnologia (ITEC), Universidade Federal do Pará (UFPA), Belém 66075-110, Brazil; francilia_campos@hotmail.com; 2 Laboratório Adolpho Ducke, Coordenação de Botânica, Museu Paraense Emílio Goeldi, Av. Perimetral, 1900, Terra Firme, Belém 66077-830, Brazil; eloisa@museu-goeldi.br (E.H.d.A.A.); lidianenascimento@museu-goeldi.br (L.D.d.N.)

**Keywords:** chocolate, GC-MS, PCA, HCA

## Abstract

This study aimed to identify the volatile compounds in the fermented and dried cocoa beans conducted with three distinct inoculants of yeast species due to their high fermentative capacity: *Saccharomyces cerevisiae*, *Pichia kudriavzevii*, the mixture in equal proportions 1:1 of both species, and a control fermentation (with no inoculum application). Three starter cultures of yeasts, previously isolated and identified in cocoa fermentation in the municipality of Tomé-Açu, Pará state, Brazil. The seeds with pulp were removed manually and placed in wooden boxes for the fermentation process that lasted from 6 to 7 days. On the last day of fermentation, the almonds were packaged properly and placed to dry (36 °C), followed by preparation for the analysis of volatile compounds by GC-MS technique. In addition to the control fermentation, a high capacity for the formation of desirable compounds in chocolate by the inoculants with *P. kudriavzevii* was observed, which was confirmed through multivariate analyses, classifying these almonds with the highest content of aldehydes, esters, ketones and alcohols and low concentration of off-flavours. We conclude that the addition of mixed culture starter can be an excellent alternative for cocoa producers, suggesting obtaining cocoa beans with desirable characteristics for chocolate production, as well as creating a product identity for the producing region.

## 1. Introduction

Being among the most appreciated products in different locations in the world, chocolate is the result of a complex processing process where different physical, chemical and microbiological reactions occur, from the collection of the cocoa fruit until the final product [1,2].

In 2019, Pará state was the largest Brazilian cocoa producer, with a total of 135 thousand tons of harvested fruit expected, representing a 25% growth in five years [3]. Recently, the city of Tomé-Açu was awarded the Geographical Indication certification, being the only municipality in Pará to have it, thus allowing greater representation in the trade of products related to the fruit [4].

The processing of cocoa beans has well-defined stages: harvesting, breaking and opening the fruit, fermentation, drying, roasting, grinding and refining, conching and tempering. Among the aforementioned stages, fermentation is one of the essential stages, since it is there that the formation of the precursors of chocolate aroma and flavour occurs. It is a natural and spontaneous biological process in which there is the participation of a microbial consortium, in which groups of yeasts, lactic and acetic acid bacteria [5,6].

Several studies in the literature report the performance of a wide variety of yeasts, lactic and acetic bacteria during fermentation in different locations around the world [7,8]; however, in the Amazon, these studies are still scarce and few studies see reporting the cacao fermentative microbiota [9,10,11].

The literature reports several studies using starter cultures for the fermentation of cocoa beans and subsequent manufacture of chocolates [12,13,14,15]. The chemical compounds formed during fermentation by the different microbial groups active in the process are responsible for the characteristic aroma and flavour of chocolate. Esters, ketones, alcohols and aldehydes are responsible for the design of fruity, floral and sweet aromas formed during the fermentation process by different yeast species, such as *Pichia kudriavzevii*, *Saccharomyces cerevisiae* and *Candida tropicalis*, for example [16,17].

Recent studies have used yeast starter cultures and were able to elucidate the importance of these microorganisms in the formation of volatile compounds in fermented almonds, thus providing almonds with desirable flavour and flavour characteristics to chocolate.

The use of *Saccharomyces cerevisiae* and *Torulaspora delbrueckii* mixed inoculum were responsible for the formation of higher amounts of alcohols and esters, responsible for notes of floral, fruity and sweet aromas [15]. The same behaviour was registered by Moreira et al. [18] when using mixed inoculum of *S. cerevisiae*, *Lactobacillus plantarum* and *Acetobacter pasteurianus* and in the study by Pereira et al. [19] with inoculants of variations of *Pichia kudriavzevii* that were also capable of accelerating fermentation on a laboratory scale.

Recent research papers with inoculants of the yeast species *S. cerevisiae* and *P. kudriavzevii* have been reporting numerous benefits for the quality of fermented cocoa beans such as obtaining almonds with fruity, sweet, floral aromas, reduced levels of acidity, increased phenolic compounds and methylxanthines levels (antioxidant and metabolic capacity) and, above all, capacity to reduce the time of the seed fermentation process, which on a large scale can favour greater productivity for the producer of the fruit [18,19,20,21,22]. In this study, we aimed to identify the volatile compounds, for the first time, formed in the on-farm fermentation of Amazonian cocoa beans, from the use of *Saccharomyces cerevisiae* and *Pichia kudriavzevii* starter cultures, through GC-MS analysis.

## 2. Results and Discussion

### 2.1. Moisture Determination of Fermented and Dried Cocoa Beans

The moisture values in each treatment were 5.70% (control treatment—CT), 5.65% (*S. cerevisiae* inoculum—ST), 5.45% (*P. kudriavzevii* inoculum—PT) and 5.55% (1:1 inoculum—SPT), showing a statistical difference between them (Tukey’s test, *p* < 0.05) (Table 1). All the cotyledons samples of fermented and dried cocoa beans were below 7% (important to prevent insect attack) and below 8% (important to prevent mould proliferation) [23]. Studies report the importance of low moisture values to promote the Maillard reaction along with protein levels [24,25,26].

### 2.2. Profile of Volatile Compounds in Fermented and Dried Cocoa Beans

**Figure 1:**
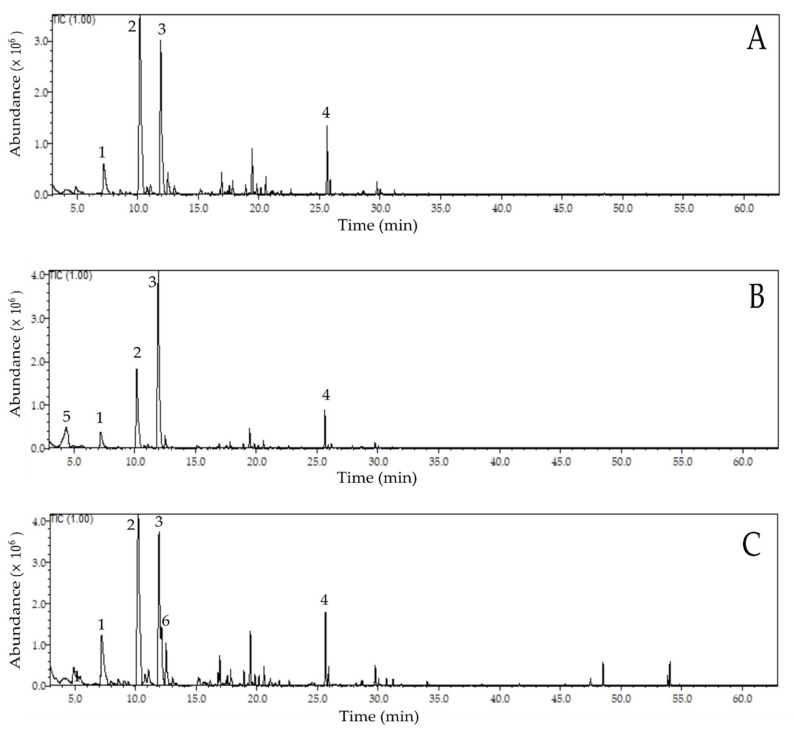

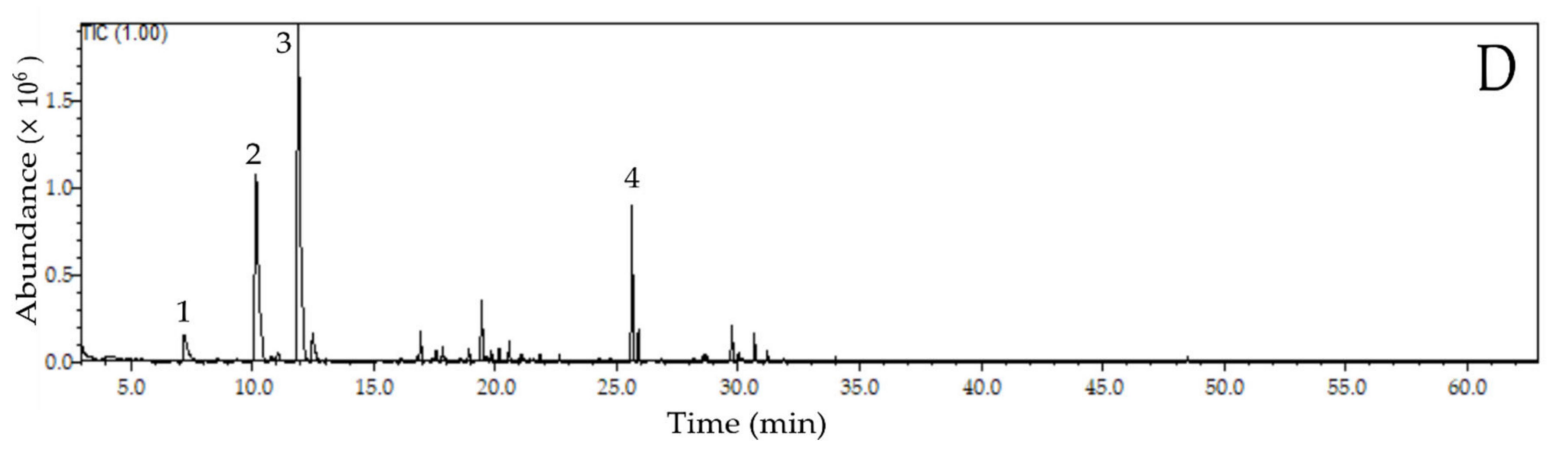
Ion Chromatograms of the GC-MS analysis on fermented and dried cocoa beans in four treatments ^1^. Notes: ^1^: CT—control treatment (**A**); ST—Saccharomyces cerevisiae inoculum (**B**); PT—Pichia kudriavzevii inoculum (**C**) and SPT—S. cerevisiae and P. kudriavzevii inoculum (1:1), (**D**). (1)—Benzaldehyde; (2)—Phenylacetaldehyde; (3)—Tetramethylpyrazine; (4)—Isoamyl benzoate; (5)—Isovaleric acid; (6)—2-Nonanone.

With chromatogram analysis (Figure 1A–D) and comparison with literature data, we identified thirty-six volatile compounds in cocoa beans in this study classified into seven distinct classes: acids, alcohols, aldehydes, ketones, esters, pyrazines and alkanes (Table 2). There was a total of 91.87% of identification in the control treatment (CT), 95.03% in the treatment with *S. cerevisiae* (ST), 93.45% in the treatment with *P. kudriavzevii* (PT), and 94.44% in the treatment with the mixed inoculum (SPT).

The acids are generally associated with the result of bacterial metabolism (lactic and acetic bacteria) developed during cocoa fermentation. The presence of acids in large quantities in cocoa beans is not desirable due to the attribution of undesirable notes of sweat, vinegar and rancidity to the quality of chocolate as a final product. The production of these compounds is also related to the performance of bacteria of the genus *Bacillus* sp. at the end of the fermentation process [5,27,28]. The ST treatment showed a higher acid content, with isovaleric acid responsible for 10.77% and acetic 2.09%. On the other hand, the PT treatment presented a greater variety of acidic compounds: octanoic, palmitic and oleic.

Only three different alcoholic compounds were identified in all fermentation treatments: 2-Heptanol, 2-Nonanol and 2-Phenylethanol. These compounds are the result of the metabolism of the yeast population active in the anaerobic phase of fermentation, after using the sugars present in the pulp of the cocoa fruit as a carbon source together with the microbial activity of the process [28,33,34].

*Saccharomyces cerevisiae* is one of the yeast species that is most prominent in the production of aromatic compounds, including alcohols, during cocoa fermentation. They manage to produce numerous desirable compounds to chocolate, (the alcoholic compounds of this study, for example), providing floral, fruity and citrus aromas [12,17]. The PT treatment produced about four times more alcohols than the other treatments, with 2-Nonanol gaining prominence, which gives citrus notes to the almond and has its formation associated with also *S. cerevisiae* [31].

The amount of 2-Phenylethanol was relatively low in the CT, ST and PT treatments, as reported in the study by Tuenter et al. [32]. The formation of this compound is the result of yeast activity during fermentation thanks to the action of glycosidase in the precursor connections of the fruit pulp aromas, and also by the conversion of the amino acid phenylalanine and it is expected to obtain chocolates with floral notes [32,35].

Five aldehydes were identified in our study: 2-Phenylbut-2-enal, Dodecanal, 5-Methyl-2-phenyl-2-hexenal and in greater quantities of Benzaldehyde and Phenylacetaldehyde. These compounds are the results of the metabolism of different yeast species at the beginning of fermentation, such as *Galactomyces geotrichum* [17].

Control fermentation (CT) and the treatment with *P. kudriavzevii* inoculum (PT), showed the highest total amount of aldehydes, showing that the population of *P. kudriavzevii* present, is capable of producing good quantities of these compounds, which has already been confirmed in laboratory scale fermentations by Pereira et al. [19]. However, the excessive amount of Benzaldehyde must be monitored to avoid the production of bitterness notes [35,36]. The same behaviour was observed in relation to ketones, where the greatest amounts of Acetophenone and 2-Nonanone stand out in the fermentation with the PT inoculum, which can confer almonds with floral and sweet notes [13,36].

A greater variety of esters can be seen in the control fermentation (CT); however, the mixed inoculation fermentation (SPT) has the highest concentrations, accounting for 13.39% of its total followed by 11.09% (CT), 7.79% (PT) and 4.74% (ST). The esters class is strongly related to almonds of good sensory quality, as they provide desirable aromatic notes of roses, flowers and fruity notes, as is the case of 2-Phenylethyl acetate and Isoamyl benzoate, which are products of yeast metabolism active in the fermentation [27,29]. In general, yeasts are relevant producers of esters during cocoa fermentation [14].

The metabolic method of production of phenylacetaldehyde was elucidated from the use of aromatic enzymes such as aminotransferase, which can convert amino acids (tyrosine, phenylalanine and tryptophan) in the stage of transamination of these compounds. These activities have been associated with lactic acid bacteria *Lactobacillus brevis* and *Lactobacillus plantarum* [37], the last of which is widely found in cocoa fermentations performed in the Amazonian region [11].

Only two types of pyrazines were identified in this study: 2,3,5-Trimethylpyrazine (only in CT fermentation) and Tetramethylpyrazine (in all treatments). The ST and SPT treatments showed the highest concentrations of Tetramethylpyrazine in fermented and dried cocoa beans. This factor is of great importance for the food industry, as these compounds confer typical notes of chocolate [6,38]. On the other hand, it is suggested to monitor the excessive amount of these compounds to avoid unpleasant notes of roasted product.

The formation of pyrazines is directly related to Strecker degradation begins in the drying process of the cocoa beans provided also by the action of the enzymes β-glycosidases, proteases, lipases and amylases, produced by bacteria of the genus *Bacillus* sp. [11,26,27,36,39]. Some parameters are decisive in the presence or absence of pyrazines in cocoa beans, such as, for example, as techniques and fermentation time, fruit maturation stage, cocoa variety, seed storage and chocolate processing [15].

In a recent study [40], the researchers elucidated the significant influence that starter cultures of yeast species *Candida parapsilosis*, *Torulaspora delbrueckii* and *Pichia kluyveri* had on the formation of and increase in flavour precursor amino acids (participants of the Maillard reaction) throughout fermentation. The authors highlight the high capacity of production of extracellular proteolytic enzymes due to the metabolism of these yeast species, with the ability to act inside the cotyledon providing a higher concentration of free amino acids in cocoa beans.

*Saccharomyces cerevisiae* and species of the genus *Pichia* are frequently identified in cocoa fermentation in several locations [7,8]. These species and several others are linked to processes that are essential in the first hours of cocoa fermentation, such as: consumption of citric acid present in the pulp; increasing the pH of the medium and favouring the development of the bacterial community; ethanol production at low oxygen concentrations; production of aromatic compounds desirable to chocolate such as ethers, aldehydes and ketones; and production of pectinolytic enzymes that favour the release of fermentable sugars from the pulp, making them available for fermentation [2,5,41].

The conversion of pulp carbohydrates into ethanol by the yeast’s metabolic activity is essential for the formation of chocolate compounds, since the ethanol formed is oxidized to acetic acid by acetic bacteria, with an increase in temperature causing the embryo to die and, thus, triggering several chemical and structural reactions that allow the formation of other flavour and flavour precursor compounds and chocolate [5,12].

*Saccharomyces cerevisiae* is a species of yeast that is widely studied in the application of inoculants in cocoa fermentation, due to its high capacity for assimilation of pulp sugars (mainly glucose and fructose), satisfactory production of volatile compounds desirable to chocolate, production of toxins capable of inhibiting some yeasts not tolerant to ethanol [2,12].

The interaction of *S. cerevisiae* and *P. kudriavzevii* was recently reported in the study by Chagas Junior et al. [20], which proved the good relationship between the two species in foreign cocoa fermentations, providing almonds with higher levels of phenolic compounds, pH and lower levels of acidity, methylxanthines, acidity and putrefactive amines, thus showing itself as an excellent alternative in the production of good quality almonds.

In our study, a good interaction was observed between these two species in relation to the production of desirable volatile compounds for cocoa beans what can highlight the production of good quality almonds for the local production of chocolate, since the production of chocolates in Pará state is still in the beginning, but its recognition is already noticed in several nations that appreciate this product. The city of Medicilândia, for example, is the largest representative of the State abroad, having several higher quality product certificates, placing Pará in international evidence.

### 2.3. Multivariate Analysis (PCA and HCA) of Volatile Compounds in Cocoa Beans Fermented and Dried Produced with Different Yeast Inocula

Finally, we performed the PCA and HCA to check and group the fermented and dried almonds in this study according to the amount of the identified volatile compound content. Previous studies have already demonstrated the importance of this analysis in cocoa fermentation in order to establish the identity of different fermentation techniques, fruit storage, drying and roasting at different temperatures and the influence of starter cultures to the process [20,27,29,42].

In order to better understand the importance of the four fermentation treatments used, we chose to analyse the volatile compounds in their entirety according to their chemical classes, for a better visualization of the results and better understanding.

**Figure 2:**
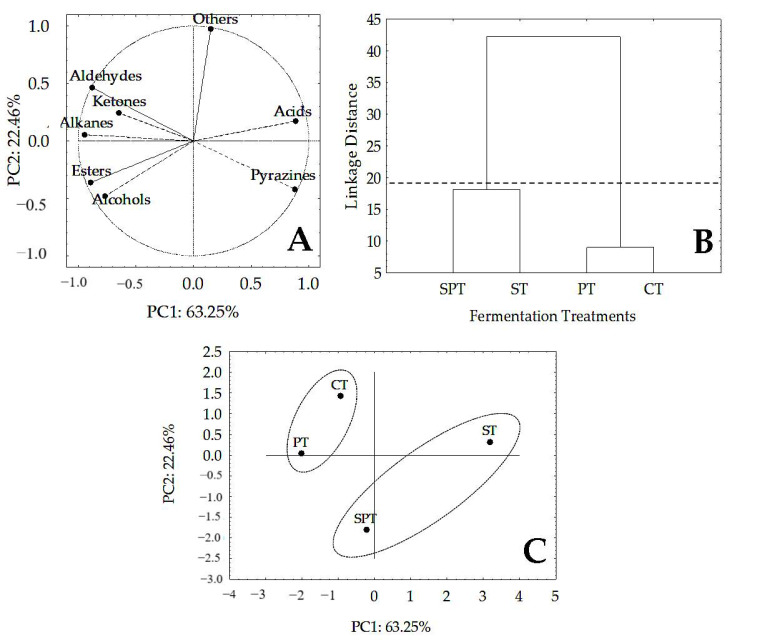
Principal Component Analysis (PCA) and Hierarchical Cluster Analysis (HCA) of volatile compounds in dried and fermented cocoa beans with different starters ^1^. Notes: ^1^: CT—control treatment, ST—*Saccharomyces cerevisiae* inoculum, PT—*Pichia kudriavzevii* inoculum; and SPT—*S. cerevisiae* and *P. kudriavzevii* inoculum (1:1). Principal Component Analysis (PCA) (**A**,**C**). Hierarchical Cluster Analysis (HCA) (**B**).

The sum of the first two components (PC1 + PC2) accounted for 85.71% of the variance of the main data (Figure 2A). Of all the chemical classes identified, esters and aldehydes were the most affected by the yeast inocula in the four fermentation treatments. The formation of aldehydes was strongly correlated (r = −0.95) with sufficient concentrations of pyrazines, which are also formed by the aldol condensation route in cocoa fermentation as well as the formation of esters from the condensation of alcohols and acids (mainly acetic acid), forming ethyl esters and acetates compounds, respectively [34]. This correlation was strongly negative between esters and acids (r = −0.97) and strongly positive between esters and alcohols (r = 0.75), elucidating the amounts of these compounds formed in this study.

With the analysis of HCA it was possible to classify the fermented and dried cocoa beans in two distinct groups (Figure 2B,C): group 1—characterized by the fermented almonds in the ST and SPT treatments that had higher concentrations of acids and lower alcohols, ketones and esters and higher amounts of pyrazines; and group 2—fermented and dried almonds in CT and PT treatments, which are characterized by having the lowest concentrations of acidic compounds and high concentrations of desirable compounds in chocolate, such as aldehydes, ketones, alcohols and esters. In this group, pyrazine values remain satisfactory. On the other hand, there is a need to monitor the content of alkanes, which are considered off-flavors [31].

It was possible with the multivariate analysis to notice that the treatments with *Pichia kudriavzevii* inocula provided the formation of higher levels of desirable compounds for cocoa beans for the production of good quality chocolate.

## 3. Materials and Methods

### 3.1. Material

Cocoa beans, fermented with *Saccharomyces cerevisiae* (Genbank KY794742) and *Pichia kudriavzevii* (Genbank KY794725) by Chagas Junior et al. [20] in Tomé-Açu city, Pará state, Brazil (02°28′41.3″ S and 48°16′50.7″ W) in September of 2017, were used in this research.

### 3.2. Fermentation Assay, Drying Process and Moisture Determination

Four fermentation treatments were carried out being one without the addition of inoculum (control treatment—CT) and three treatments with different inocula: *Saccharomyces cerevisiae* inoculum (ST); *Pichia kudriavzevii* inoculum (PT) and fermentation with the 1:1 addition of both species (SPT). All treatments were performed in triplicate (*n* = 3) according to the standard procedures established by the local producer with no external interference, followed by seven days of fermentation for CT, ST and PT treatments and six days for SPT treatment once the producer noticed the completion of the process [20]. All the fermentations were carried out in wooden boxes with a dimension of 0.12 m^3^, each containing 90 kg of cocoa beans in each fermentation treatment.

At the end of the process, the fermented cocoa beans were packed in sterile polyethylene bags under freezing (−18 °C) for subsequent artificial drying in an air circulation oven (DeLeo, Porto Alegre, RS, Brazil) at 35 °C until at a constant moisture of 6% [43].

The shells and embryo of the fermented and dried cocoa beans of each treatment were removed and the cotyledons were ground in an analytical mill (model A11B, Ika, Staufen, Germany) for moisture determination. This process was performed by direct analyse on an infrared moisture analyser (model IV2500, Gehaka, SP, Brazil). The analysis was performed in triplicate.

### 3.3. Gas Chromatography-Mass Spectrometry (GC-MS) in Fermented and Dried Cocoa Beans

Samples of fermented and dried cocoa beans (20 g) of each fermentation treatments were subjected to the simultaneous distillation/extraction process for 2 h, using pentane (Sigma-Aldrich, St. Louis, MO, USA) as a solvent [44,45,46]. The volatile concentrate obtained was analysed by GC-MS (model QP-2010 Plus, Shimadzu, Tokyo, Japan), equipped with a DB-5MS column (30 m × 0.25 mm × film thickness = 0.25 µm). The oven temperature was adjusted from 60–250 °C, using a ramp of 3 °C/min, injector temperature of 250 °C. Helium gas was used as a mobile phase, with a flow rate of 1.2 mL/min. An electron ionization mass spectrometer (model GC-2010A, Shimadzu, Tokyo, Japan) at 70 eV with the ion source temperature and other parts at 220 °C was used. Quantitative analysis of the chemical constituents was performed by peak-area normalization using a flame ionization detector (FID—Shimadzu, QP 2010 system) under the same conditions as GC-MS, except that nitrogen was used as a mobile phase. The components were identified based on the retention index (RI), which was calculated using the retention times of a homologous series of n-alkanes (C8–C40, Sigma-Aldrich, St. Louis, MO, USA). The pattern of fragmentation observed in the spectra was compared with existing data in the system library and with data from the literature [44,45,46].

### 3.4. Statistical Analysis

The means of the moisture determination were verified according to the Analysis of Variance (ANOVA) and compared with Tukey’s test at 5% significance. The Principal Component Analysis (PCA) was performed to group the volatile compounds identified and quantified in the cocoa samples in all treatments used (CT, ST, PT, SPT). The sum of the compounds of each chemical class was considered as the active variables. For Hierarchical Cluster Analysis (HCA), the hierarchical tree was obtained taking into account the same groups of active variables as the PCA, based on the Euclidean distances (Ward’s method) for the grouping. The statistical analyses were performed with Statistica 7.0 software (StatSoft Inc., Tulsa, OK, USA).

## 4. Conclusions

In this research, we observed that inoculations with the species *P. kudriavzevii* (PT, SPT) provided the formation of a greater variety of desirable compounds for cocoa beans intended for chocolate production, such as, for example, a greater variety of aldehydes and esters (fruity floral, sweet notes) and lesser amounts of undesirable compounds (off-flavors) such as alkanes and acids, proving to be an excellent alternative for cocoa almond producers in the region. To better understand the physiological dynamics and synergy of the microbiota responsible for the development of these compounds, they must be carried out and thus establish parameters for the development of starter cultures that can be used by producers and the food industry.

## Figures and Tables

**Table 1:** Moisture (%) values * in fermented and dried cocoa beans, inoculated with different yeasts inocula: CT—control fermentation, ST—*Saccharomyces cerevisiae* inoculum, PT—*Pichia kudriavzevii* inoculum, SPT—inoculum with both species (1:1).

	CT	ST	PT	SPT
Moisture (%)	5.70 ± 0.00 ^a^	5.65 ± 0.07 ^ab^	5.45 ± 0.07 ^b^	5.55 ± 0.07 ^ab^

* in dry base. Means (± standard deviation) with different letters in the same line are statistically different (Tukey’s test, *p* ≤ 0.05).

**Table 2:** Volatile compounds (GC-MS) in fermented and dried cocoa beans with the addition of three different yeast starter cultures ^1^.

RI	Compound	CT (%)	ST (%)	PT (%)	SPT (%)	Odour Description ^2^
**Acids**						
841	Isovaleric acid	-	10.77	-	-	Sweat, rancid (off-flavour)
1173	Octanoic acid	-	-	0.38	-	Sweat, fatty (off-flavour)
1256	Acetic acid	-	2.09	-	-	Sour, vinegar (off-flavour)
1964	Palmitic acid	-	-	0.33	-	
2169	Oleic acid	-	-	1.25	-	
**Alcohols**						
894	2-Heptanol	-	-	0.32	-	Fruity, citrus, herbal
1099	2-Nonanol	-	-	3.98	2.91	Fat, green
1111	2-Phenylethanol	0.93	0.15	0.35	-	Honey, rummy, floral
**Aldehydes**						
955	Benzaldehyde	6.99	4.68	10.19	3.30	Roasted almonds, candy, burnt sugar
1040	Phenylacetaldehyde	36.50	21.79	30.78	26.87	Floral, honey
1271	2-Phenylbut-2-enal	0.45	0.26	0.56	0.70	Floral, honey, powdery, cocoa
	Dodecanal	-	-	-	0.39	
1490	5-Methyl-2-phenyl-2-hexenal	1.01	0.46	1.22	1.85	Cocoa
**Ketones**						
1062	Acetophenone	1.65	0.56	1.45	-	Flower, almond, pungent, sweet
1090	2-Nonanone	-	-	5.25	-	Fruity, sweet, waxy, green herbaceous
1119	Isophorone	0.09	-	-	-	
**Esters**						
868	Isopenthyl acetate	0.94	-	1.94	-	
1163	Benzyl acetate	-	-	0.34	-	Floral, jasmine
1169	Ethyl benzoate	0.16	-	-	-	Camomile, flower, fruity
1196	Ethyl octanoate	0.38	0.21	-	-	Fruity, floral
1255	2-Phenethyl acetate	4.36	0.53	4.21	4.23	Fruity, sweet, roses, honey, floral
1394	Isoamyl benzoate	5.09	4.00	4.51	8.95	Balsam, sweet
1594	Ethyl dodecanoate	0.06	-	0.15	-	Sweet, floral
1994	Ethyl hexadecanoate	0.10	-	1.15	0.21	
**Pyrazines**						
1000	2,3,5-Trimethylpyrazine	0.59	-	-	-	Roasted cocoa
1084	Tetramethylpyrazine	24.76	46.77	19.71	41.20	Roasted cocoa, chocolate
**Alkanes**						
1055	3-Methyldecane	-	-	1.09	-	
1200	*n*-Dodecane	1.68	0.48	1.74	1.78	Off-flavour
1264	2-Methyldodecane	0.78	-	-	-	
1280	4,6-Dimethyldodecane	1.18	-	-	-	
1300	Tridecane	0.11	-	0.16	-	Off-flavour
1400	*n*-Tetradecane	0.93	-	0.93	1.51	Off-flavour
1490	Pentadecane	0.12	0.25	0.30	0.42	Off-flavour
1711	Heptadodecane	-	-	-	0.12	Off-flavour
**Others**						
1099	Linalool	2.78	2.03	-	-	Floral
1181	Naphthalene	0.23	-	1.16	-	
	**Total (%)**	91.87	95.03	93.45	94.44	

Notes: RI: retention index. ^1^: CT: control fermentation, without addition of inoculum; ST: fermentation with *Saccharomyces cerevisiae* inoculum; PT: fermentation with *Pichia kudriavzevii* inoculum; SPT: fermentation with the 1:1 addition of both species. ^2^: These characteristics were found in the literature [6,26,27,29,30,31,32].
